# Polyphenols vs. Caffeine in Coffee from Franchise Coffee Shops: Which Serving of Coffee Provides the Optimal Amount of This Compounds to the Body

**DOI:** 10.3390/molecules29102231

**Published:** 2024-05-09

**Authors:** Regina Ewa Wierzejska, Iwona Gielecińska, Ewelina Hallmann, Barbara Wojda

**Affiliations:** 1 Department of Nutrition and Nutritional Value of Food, National Institute of Public Health NIH—National Research Institute, Chocimska St. 24, 00-791 Warsaw, Poland; bwojda@pzh.gov.pl; 2 Department of Food Safety, National Institute of Public Health NIH—National Research Institute, Chocimska St. 24, 00-791 Warsaw, Poland; igielecinska@pzh.gov.pl; 3 Institute of Human Nutrition Sciences, Department of Functional and Organic Food, Warsaw University of Life Sciences, Nowoursynowska St. 159c, 02-776 Warsaw, Poland; ewelina_hallmann@sggw.edu.pl; 4 Bioeconomy Research Institute, Agriculture Academy, Vytautas Magnus University, K. Donelaičio Str. 58, 44248 Kanuas, Lithuania

**Keywords:** polyphenols, caffeine, coffee, franchise coffee shops, coffees serving size

## Abstract

The scientific literature indicates that there is a limited number of data on the content of bioactive components in coffees consumed “on the go”. Therefore, this study examined the polyphenol and caffeine content of different types of coffee from franchise coffee shops, and the caffeine/total polyphenol ratio. The five most popular types of coffee purchased in six franchise coffee shops in Warsaw were analysed. A total of 120 coffee samples were tested. A significant positive (r = 0.7407, *p* < 0.001) correlation was found between the total polyphenol and caffeine content in all coffee types tested. Per unit volume, espresso coffee had the highest significant (*p* < 0.005) average total polyphenol and caffeine contents (232.9 ± 63.9 mg/100 mL and 198.6 ± 68.3 mg/100 mL, respectively). After taking into account the coffee’s serving size, a serving of Americano provided significantly (*p* < 0.05) the most total polyphenol (average 223.5 ± 81.5 mg), while the highest caffeine content was provided by a serving of ice latte/latte frappe (average 136 ± 57.0 mg). The most favourable ratio of caffeine to total polyphenols (0.56) was found in a serving of Americano coffee; therefore, it seems that this coffee can be considered optimal in terms of the content of both compounds. These findings demonstrate that the polyphenol and caffeine contents of coffees offered in franchise coffee shops are closely related to the serving size.

## 1. Introduction

Coffee is currently considered one of the food items that support overall health. Drinking moderate amounts of coffee on a regular basis is believed to reduce the risk of type 2 diabetes, cardiovascular diseases, some cancers, etc. [1,2,3,4]. The coffee infusion is a complex mixture of many biologically active substances, of which the components with physiological effects—polyphenols and caffeine—have attracted the most attention from researchers. Although caffeine is supposed to be responsible for improving general well-being, after drinking coffee [5,6] the caffeine intake should be kept in moderation, and in light of current knowledge, the safe intake of this alkaloid for adults is up to 400 mg per day and up to 200 mg for pregnant women [7,8]. Single doses of caffeine, up to 200 mg (about 3 mg/kg bw for a 70-kg adult), do not give rise to safety concerns [7]. Polyphenols, on the other hand, are highly beneficial dietary components with anti-inflammatory and antioxidant properties, and it is currently presumed that those found in coffee are as valuable to the body as those in vegetables and fruits [9,10]. A number of factors affect the caffeine and polyphenol content of coffee infusions, including the botanical species of coffee, growing conditions, roasting method, grind size, brewing method and the strength of the infusion [11,12,13]. All of this makes it difficult to predict the amount of these ingredients in a coffee serving, not only the one we order at a coffee shop but also the one we make every day in the same way at home. Although it is believed that there is no “perfect” coffee, the proportion of caffeine and polyphenols affects its health properties [14,15]. It is worth mentioning that the European Food Safety Authority (EFSA) considers it scientifically proven that a single intake of at least 75 mg of caffeine increases alertness, which has not been confirmed for lower doses of caffeine [16]. Therefore, from the point of view of mental performance, an overly light brew of coffee may not be the optimal choice.

Experts recommend drinking 3–5 cups of coffee per day [1,17], but scientific publications do not specify the size of a cup or a single serving, yet some people drink coffee in mugs. Only US recommendations specify the cup volume (8 oz, i.e., ~240 mL) [18]. Thus, the literature emphasises that a cup of coffee is not a sufficiently characterised measure of either the volume of coffee consumption or its components [19,20,21], and data on the caffeine content of the servings of coffee that consumers buy are scarce and need to be supplemented [6,11,22]. What is more, the content of polyphenols and caffeine in a coffee serving reported in the literature generally does not take into account either the method of brewing coffee or the serving size, which is highly relevant. Therefore, the more information there is about the composition of differently prepared coffees, the more accurately it will be possible to estimate the dietary intake of these compounds in scientific research. This is particularly crucial for coffee consumed by vulnerable populations, such as pregnant women, with careful consideration given to the specific type of coffee being consumed. Moreover, taking into account the impact of coffee components on the body, the ratio of caffeine to total polyphenols may be a useful parameter for the health properties of coffee [15].

Therefore, the aim of this study was to determine the polyphenols and caffeine content of coffee infusions available at franchise coffee shops, and then calculate the caffeine/total polyphenol ratio in order to select the coffee that is optimal in terms of both compounds. The optimal serving of coffee was considered to be coffee with a low caffeine/total polyphenol ratio, and, taking into account the position of EFSA, also containing, on average, not less than 75 mg but not more than 200 mg of caffeine [7,16].

## 2. Results

### 2.1. Polyphenol Content per 100 mL of Coffee

The total polyphenol content in 100 mL of the coffees tested averaged 101.3 mg (min–max: 21.3–343.1 mg/100 mL). Espresso coffee had the highest (*p* < 0.005) polyphenol level (Table 1). Each type of coffee showed a large variation in the content of polyphenols within the group. Cappuccino exhibited an approximately 2-fold difference, while Americano displayed a variance of more than 4-fold.

Gallic acid and chlorogenic acid were found to be the predominant polyphenols in all the coffee samples analysed. When it comes to gallic acid, significantly (*p* < 0.05) the lowest content was found in cappuccino coffee, while the highest (*p* < 0.05) was in espresso and Americano. With regards to chlorogenic acid, caffè latte/latte macchiato and ice latte/latte frappe coffee had significantly (*p* < 0.0001) the lowest content of this compound, compared with the other coffees tested (Table 1).

### 2.2. Polyphenol Content per Coffee Serving

**Figure 1:**
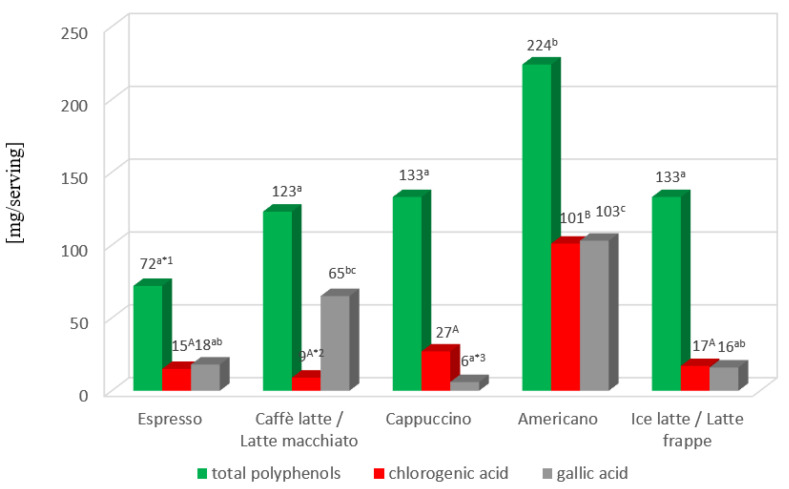
The content of total polyphenols and selected phenolic acids in coffee serving. *^1^ Statistically significant difference (*p* < 0.005) in the total polyphenols level between various coffee brew types. Values marked with different letters were significantly different. *^2^ Statistically significant differences (*p* < 0.0001) in chlorogenic acid levels between various coffee brew types. Values marked with different letters were significantly different. *^3^ Statistically significant differences (*p* < 0.05) in gallic acid levels between various coffee brew types. Values for a given compound marked with different letters were significantly different.

The total polyphenol content in a serving of the coffees tested averaged 137.0 mg (min–max: 35.4–321.1 mg). The highest statistically significant (*p* < 0.005) total polyphenol content was found in a serving of Americano coffee (Figure 1). Differences in total polyphenol level, depending on the coffee type, were 3 fold (espresso, caffè latte/latte macchiato, cappuccino and Americano) or more than 4-fold (ice latte/latte frappe).

As shown in Figure 1, a serving of Americano coffee also had the highest statistically significant (*p* < 0.05) content of gallic acid and chlorogenic acid.

### 2.3. Caffeine Content per 100 mL of Coffee

The average caffeine amount in 100 mL of coffee was found to be 79.7 mg (min–max: 20.5–370.4 mg/100 mL). Espresso coffee had the highest statistically significant (*p* < 0.0001) mean caffeine content (198.6 mg/100 mL), while ice latte/latte frappe had the lowest (43.9 mg/100 mL) (Table 1). It should be noted that the mean caffeine level of espresso coffee was about 4-times higher (*p* < 0.0001) compared to the other coffees, while there were no statistically significant differences in the caffeine content among the other types of coffees tested. Similar to polyphenols, there was considerable variability observed in the caffeine content among the different coffee groups tested. The difference between the smallest and largest amount of caffeine in the samples of most coffees was more than 3-fold, while for caffè latte/latte macchiato and Americano—it was more than 4-fold.

### 2.4. Caffeine Content per Coffee Serving

**Figure 2:**
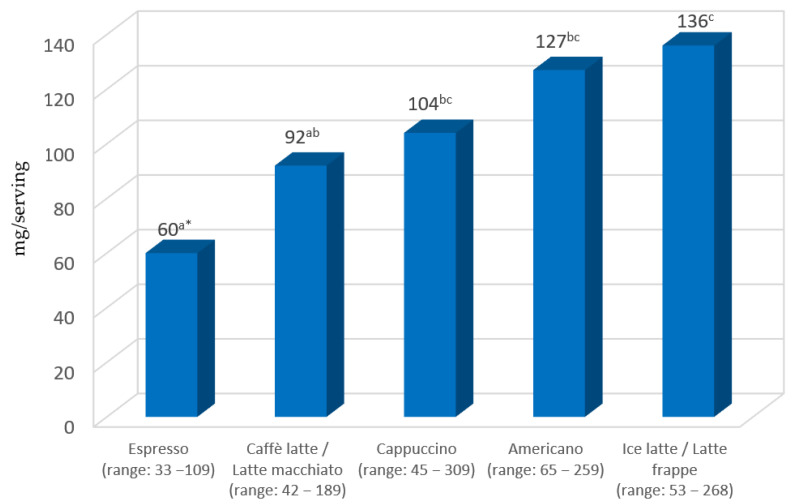
Caffeine content in coffee servings. * Statistically significant difference (*p* < 0.05) between various coffee brew types. Values marked with different letters were significantly different.

The average caffeine content per serving of coffee was found to be 103.7 mg (min–max: 32.5–309.4 mg). The order of coffees in terms of caffeine content per serving was opposite compared to caffeine level per 100 mL of coffee. As shown in Figure 2, a serving of espresso provided statistically significantly (*p* < 0.05) the least caffeine, while a serving of ice latte/latte frappe was the most (*p* < 0.05). In all types of coffees tested, there was a large variation in caffeine content per serving (3- to 4-fold for espresso, caffè latte/latte macchiato and Americano, and almost 7-fold for cappuccino and ice latte/latte frappe).

### 2.5. Relationship between Polyphenol and Caffeine Content

**Figure 3:**
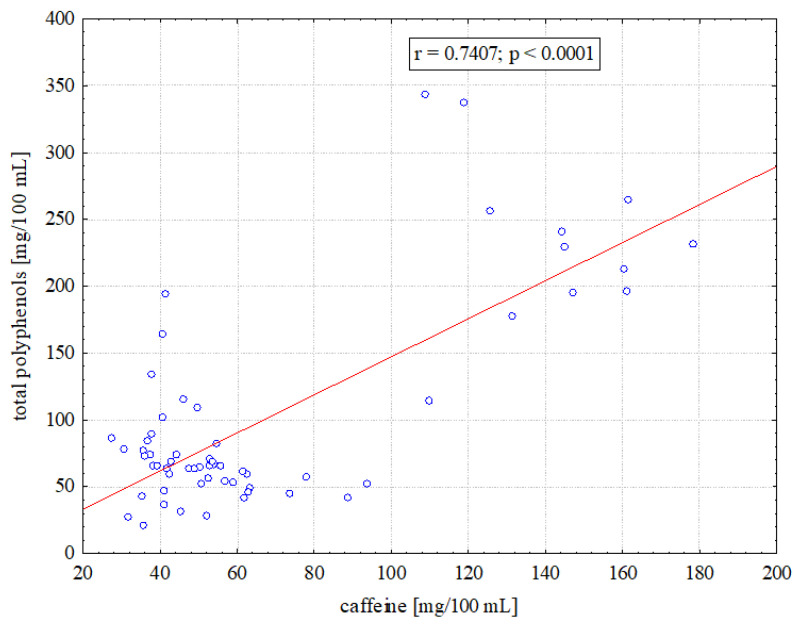
Correlation between total polyphenol and caffeine levels in coffee.

Statistical analysis showed a strong positive correlation (r = 0.7407, *p* < 0.001) between the content of total polyphenols and caffeine in all coffee types (Figure 3). Furthermore, a significant correlation was found between the caffeine level and the content of two out of the ten polyphenols examined, specifically gallic acid (r = 0.3902, *p* < 0.05) and caffeic acid (r = 0.6774, *p* < 0.001). For the other polyphenolic compounds tested, the correlations were much weaker and were not statistically significant.

The lowest significant (*p* < 0.05) caffeine/total polyphenol ratio was found for Americano and espresso coffee, while the highest (*p* < 0.05) was for ice latte/latte frappe (Table 2). Therefore, also taking into account the average caffeine content in a serving of Americano (127 mg), which provides the alertness-increasing dose of caffeine (minimum 75 mg), a serving of this coffee can be considered optimal in terms of both analysed components.

In terms of chlorogenic acid, Americano coffee demonstrated the lowest ratio, whereas caffè latte/latte macchiato was the highest. When it comes to gallic acid, the ratio of caffeine to this compound was lower for caffè latte/latte macchiato, espresso and Americano compared with cappuccino and ice latte/latte frappe. The aforementioned values were statistically significant (*p* < 0.05).

## 3. Discussion

Polyphenols and caffeine are natural components of coffee, and as with all substances present in foods of plant origin, their content is subject to significant fluctuations. When it comes to polyphenols, it is sometimes assumed that the total polyphenols amount to 200 mg/100 mL of coffee, and there are 300 mg in a serving [23,24], but there are also data indicating that the polyphenol content in a serving of coffee is much higher (500–1200 mg) [11,14]. This shows a high variability in the polyphenol amount, which depends on numerous factors. In our study, the total polyphenol content was lower and in 100 mL of coffee it was on average 101 mg, while in a serving of coffee, which for most coffees was higher than 100 mL, the average was 137 mg. When relating our results to the literature data, it should also be borne in mind that published studies differ in terms of the analytical methods used (including the limit of quantification), and the manner in which results are presented (such as average/mean or median).

Comparison of the content of individual polyphenolic compounds is difficult, as published studies deal with different groups of polyphenols. The main coffee polyphenols are chlorogenic acids (CGAs), including caffeoylquinic acids (CQAs) as a subclass of CGAs [25,26,27]. According to the literature data, the content of CGAs in a cup of coffee ranges from 20 mg to 675 mg [26] or from 70 mg to 350 mg [23,28]. The content of CGAs in a serving of espresso coffee determined in our study (mean 15 mg) is relatively small compared to the results obtained by other authors. In a study by Crozier et al. [25], Scottish espresso from Costa Coffee had an average of 227 mg of total CQA, but from Starbucks only 24 mg. In a study by Ludwig et al. [21], Scottish, Spanish and Italian espresso from coffee shops had a median total CQA in a serving of coffee of 59 mg, 142 mg and 46 mg, respectively. In Americano coffee, the CGA content we found (mean 101 mg) is comparable to the results of a Korean study [15], in which the total CGAs in a serving of coffee purchased from coffee shops averaged 99.4 mg. In the case of cappuccino coffee, the CGA content (27 mg per serving) fell within the range of CQA’s content for coffee purchased in Scottish coffee shops, where the mean content of these compounds ranged from 23 to 135 mg per serving, depending on the type of coffee shop [21].

With regard to caffeine, many authors assume that a standard cup of coffee (~240 mL) contains 100 mg [29,30,31], 107 mg [32] or 135 mg [33] of caffeine. Still, other authors believe that an average cup of coffee is 150 mL in size and provides 80–90 mg [7,34,35], 60 mg [36] or only 50 mg [37] of caffeine. In our study, without taking into account the coffee type, an average serving of coffee from franchise coffee shops (192 mL) contained 104 mg of caffeine. As far as coffee type is concerned, the caffeine content of a serving of coffee (22–420 mL) averaged between 60 mg and 136 mg and increased as the serving size increased. According to a US study [34], a coffee serving, depending on its type, provides from 58 mg to 259 mg of caffeine, and similarly, the largest differences stem from the serving size (30–473 mL). These data support the view that assuming a constant caffeine content for a cup of different coffees may be either overestimated or underestimated, making it difficult to assess caffeine intake, as well as compare it to a safe level.

One of the most widely consumed coffees is espresso. In Poland, it is consumed by more than 90% of coffee drinkers [23], so data on the caffeine content in this coffee type seems particularly useful. Espresso, compared to other coffees, is considered a strong coffee, i.e., a coffee with a high caffeine content per unit volume, which is due to the use of a large amount of ground coffee relative to the amount of water [6,11,14]. This was also confirmed by our study. However, it should be noted that the study did not cover coffees brewed by other methods, such as drip coffee or Turkish coffee. From the consumer’s point of view, of much greater importance is the amount of caffeine supplied to the body with a coffee serving, and with this approach, the strength of the coffees studied took the reverse order compared with 100 mL. Espresso coffee, served in portions several times smaller than other coffees (an average of 31 mL in our study), contained the least caffeine (mean 60 mg). There have been no studies in Poland to date on the caffeine content of “takeaway” coffees, so the authors cannot compare their results at the local level. In light of the world literature, the mean caffeine content we found is highly consistent with many, but not all, of the data. O‘Keefe et al. [38] cite that espresso usually has about 60 mg per shot. The same result was obtained by McCusker et al. [34] in a serving of American espresso from coffee shops (58 mg of caffeine in a 30 mL serving), which also coincides with the USDA Food Composition Databases [39] and, according to which, a cup of espresso (1 oz; ~30 mL) contains an average of 63 mg of caffeine. A considerably higher content of this ingredient, compared with our result, was found in Scottish espresso (median 140 mg/serving, median serving 43 mL) [25]. However, it is worth noting that among 20 different outlet coffee shops in Glasgow where the authors of the aforementioned study purchased espresso, only two—Starbucks and Costa Coffee—overlapped with our study. Ludwig et al. [21], analysed Scottish, Italian and Spanish espresso from coffee shops and found comparable amounts of caffeine in these coffees (median 100 mg, 102 mg and 116 mg, respectively), but their results are higher than in our study. Other authors report a lower caffeine content. According to Crozier et al. [25], a cup of classic espresso (30 mL) supplies 30–50 mg of caffeine, while according to Heckman et al. [29]—40 mg. All studies reveal significant differences in the caffeine content of an espresso serving, depending on the coffee shops. The values vary more than 6-fold [25], more than 5-fold [21], and—in our study—more than 3-fold. This may not be surprising, since more than 2-fold differences were found even in the same type of coffee, purchased from the same coffee shop for six consecutive days [34].

When it comes to Americano coffee, it is worthwhile to compare the caffeine content found (an average of 127 mg/serving) with the results of a study by Jeon et al. [15], who bought Americano coffee at eleven coffee shops, four fast-food restaurants and three bakery stores. The caffeine content of coffee from coffee shops averaged 166 mg/serving, from fast-food restaurants—108 mg/serving, and from bakery stores—94 mg/serving. Considering only coffees from coffee shops and fast-food restaurants, i.e., places included in our study as well, the mean caffeine content in both studies is highly consistent (137 mg/serving vs. 127 mg/serving). According to van Dam et al. [40], a serving of Americano from coffee shops (12 oz; ~340 mL) contains 150 mg of caffeine, while according to the USDA Food Composition Databases—94–150 mg [39]. Slightly smaller amounts in a serving of Americano (8 oz; ~240 mL) are reported by Mitchell et al.—63–126 mg of caffeine [41]. It is worth mentioning that Americano coffee offered in coffee shops (referred to as American coffee imitation) is espresso-based, often including double espresso. In our survey, the use of double espresso was declared by baristas from three out of six coffee shops, which, also considering the much larger serving size of such coffee, explains the higher caffeine content compared with espresso.

In the case of cappuccino, the mean caffeine content in our study (104 mg) is congruent with data reported by Mattioli and Farinetti—110 mg [42]. Cappuccino coffee purchased in coffee shops in Scotland had a higher caffeine content, with a median of 180 mg [21].

With regard to other types of coffee offered in coffee shops, there is little data in the literature. Caffè latte, according to the USDA Food Composition Databases [39], contains as much caffeine as cappuccino (mean 86 mg/serving), which coincides with the results of our study (92 mg). In Norway, caffè latte and cappuccino are also assumed to contain the same amount of caffeine but at a much lower level—21 mg/100 mL [43], which would be 50 mg for a 240 mL serving. A serving of ice latte/latte frappe coffee had the highest caffeine content in our study (mean 136 mg), but note that the serving size was expressed as the volume of the coffee alone after separating the ice immediately after purchasing the coffee (coffee with ice had up to 40% more volume than coffee without ice). According to US charts, a serving (8 oz; ~240 mL) of iced coffee provides less caffeine (74 mg), but there is no information on the preparation method, which constitutes a factor that determines the content of this ingredient [39]. It should be noted that in our study, in three out of the six coffee shops, ice latte/latte frappe was prepared based on double espresso, which may explain the higher caffeine content.

When considering the caffeine content of coffees from franchise coffee shops, it is worth relating it to the dose that benefits mental performance. According to EFSA [16], in most people, an intake of 75 mg of caffeine in a single dose is necessary for increased alertness, so such an effect cannot be produced by either light coffee infusions or too small a coffee serving. Given the average caffeine content in the coffees tested, this would apply to a serving of espresso, which may come as a surprise to many, since such coffee is generally considered strong. However, taking into account the caffeine content of individual coffee samples, a dose lower than 75 mg/serving was found in 36% of all coffee samples tested (in 75% of espresso coffee samples, 50% of caffè latte/latte macchiato, 33% of cappuccino, 17% of Americano and 8% of ice latte/latte frappe samples).

Our study showed a strong correlation between the level of total polyphenol and caffeine in all coffee types tested. This means that coffee supplying a large amount of caffeine also contains a high level of polyphenols. Polyphenols are compounds with beneficial effects on the body and it is polyphenols that are mainly responsible for the health-enhancing effects of coffee [9,44]. Thus, it would be fair to say that for people who like coffee and tolerate it well, coffee is a much better choice than caffeinated sodas. For the average consumer, and especially for people who should limit their caffeine intake, such as pregnant women, the most suitable option would be coffee containing more polyphenols than caffeine. The low caffeine/total polyphenols ratio means that the level of polyphenols is relatively high compared to the caffeine level. The most favourable ratio of caffeine to total polyphenols in our study was found for Americano coffee (mean 0.56), and the least favourable was for ice latte/latte frappe coffee (mean 1.17). Therefore, coming back to the original question in the title of the article about the optimal serving of coffee, in terms of polyphenols and caffeine, it should be stated, that for an average consumer, it would be a serving of Americano. It seems that espresso which also had a low caffeine-to-polyphenol ratio (0.65) cannot be considered optimal coffee because the average caffeine content in a serving of this coffee (60 mg) was lower than alertness-increasing dose of caffeine (minimum 75 mg). Moreover, the minimal caffeine content found in the Americano coffee samples (65 mg) is the closest to the dose of 75 mg, compared to the minimal caffeine content of espresso (33 mg). This means that the risk of buying Americano containing very little caffeine is low. Since the total polyphenol content consists of a whole range of compounds, the calculated ratio of caffeine to individual polyphenols takes a value above one. The ratio of caffeine to CGA (chlorogenic acid) for espresso averaged 4.3, and for Americano 1.5, indicating that the amount of caffeine far exceeds the amount of CGA. Also, in Scottish espresso from coffee shops, purchased four times, the ratio of caffeine to CGAs (chlorogenic acids) exceeded the value of 1 in each case, ranging from 1.5 to 10.4 depending on the coffee shop [21]. In Americano coffee from franchise coffee shops in the Republic of Korea, this ratio averaged 2.1 [15].

A limitation of our study is the lack of detailed information on how the coffee is prepared (the amount of coffee beans used, brewing time, and, for coffees with milk, the amount of milk used). The differences between the smallest and the largest content of polyphenols and caffeine confirm the high variability of these components, but the authors are unable to identify the most significant factor. Data obtained from coffee shops show that four of them used only Arabica coffee, and two used a mix of Arabica and Robusta, which could theoretically have contributed to the resulting variability. However, the purpose of the study was not to analyse individual factors affecting coffee composition but to estimate polyphenol and caffeine content from a consumer point of view. One of the advantages of our study was that we bought coffee several times in the same coffee shops located in different parts of the city, which, in addition to the fact that the coffee was prepared by different staff, also increased the chances of including different batches of coffee beans. Furthermore, the study included five types of coffee most frequently chosen by Poles from six popular franchise coffee shops located throughout Warsaw. In Poland, there have been no studies so far on the polyphenol and caffeine content of coffees bought in coffee shops; therefore, with the growing popularity of such establishments, the data obtained may serve to estimate the dietary intake of the components in question more accurately.

## 4. Materials and Methods

### 4.1. Study Material

Five types of the most popular coffees in Poland [45] were selected for the study. These types included espresso, cappuccino, caffè latte/latte macchiato, Americano and ice latte/latte frappe. No sugar, cream, etc. was added to the coffee. The samples of coffee were purchased at six different franchise coffee shops in Warsaw, including four popular chain coffee shops (Costa Coffee, Starbucks, Tchibo, Green Caffè Nero), one fast-food restaurant—McCaffé (McDonald’s) and one outlet located at BP gas stations—Wild Bean Cafe. In order to obtain a comprehensive and reliable assessment of caffeine and polyphenol levels, which depend on the type of coffee bean, among other factors, samples were collected four times (July, September, October and November 2021) from the same establishments. The caffeine content was determined in all samples purchased, while polyphenol levels were determined only in samples taken in the months of July and September. A total of 120 coffee samples were tested.

The purchased coffee samples were consistently delivered to the laboratory of the Department of Food Safety at the National Institute of Public Health NIH—National Research Institute. Here, average laboratory samples were meticulously prepared. One portion of these samples underwent caffeine content analysis on the same day, while the remainder was subsequently transported to the Department of Functional and Organic Food at Warsaw University of Life Sciences. In the laboratory, the total polyphenol content, as well as the levels of specific phenolic acids and flavonols, were meticulously determined.

### 4.2. Total Polyphenol Content Determination

The total polyphenol content, expressed as gallic acid equivalent, was performed using the Folin colorimetric method. The sample preparation for testing followed the procedure described by Singleton et al. [46]. Briefly, 5 mL of coffee brew sample was measured into a 250 mL beaker and water was added. Extraction was carried out in an ultrasonic bath (30 °C, 6000 Hz, 20 min). Subsequently, the sample was subjected to vacuum filtration and further diluted at a ratio of 1:15 with water. From this prepared solution, 1 mL was transferred into a 50 mL volumetric flask. Then, to this flask, 2.5 mL of Folin-Ciocalteu reagent (F-C) and 5 mL of 20% sodium carbonate were added, followed by topping off with water. The sample was left to incubate under room temperature, shielded from light, for a period of 45 min. Afterwards, the absorbance at a wavelength of λ = 750 nm was measured using a spectrophotometer (Helios γ, Thermo Scientific, Waltham, MA, USA).

### 4.3. Selected Phenolic Acid and Flavonol Content Determination

The quantitative and qualitative analysis of polyphenolic compounds was performed using the high-performance liquid chromatography (HPLC) method described earlier by Król et al. [47]. Briefly, a 3 mL sample of coffee brew was combined with 5 mL of 80% methanol. The mixture was then mechanically shaken and subjected to extraction in an ultrasonic bath (30 °C, 10 min). Following extraction, the sample was centrifuged (5 °C, 3780× *g*, 10 min) and was subsequently subjected to chromatographic analysis (HPLC-DAD using two LC-20AD pumps, a CBM-20A controller, an SIL-20AC column oven, and UV/Vis SPD-20 AV, and SPD-M20A spectrometers; Shimadzu, Kyoto, Japan).

Chromatographic separation was carried out on a Phenomenex Fusion-RP 80A column (Torrance, CA, USA, 250 mm × 4.60 mm). The mobile phase consisted of two components: (A) 90% water and 10% acetonitrile, and (B) 45% water and 55% acetonitrile, both with a pH of 3.0. The applied gradient program is detailed in Table 3. The injection volume was set at 100 μL, with the sample temperature maintained at 30 °C, and the column temperature also held at 30 °C. UV detection was performed at wavelengths: λ = 250 nm for flavonoids (quercetin-3-*O*-rutinoside, kaempferol-3-*O*-glucoside, quercetin, quercetin-3-O-glucoside, kaempferol, epigallocatechin) and λ = 370 nm for phenolic acids (gallic, chlorogenic, caffeic, salicylic).

### 4.4. Caffeine Content Determination

The caffeine content in coffee samples was determined using high-performance liquid chromatography with a diode array detector (HPLC-DAD) according to an in-house test procedure. The study sample was prepared as previously described [48]. Briefly, a coffee brew ranging from 2.5 to 7.5 mL (depending on the expected caffeine content) was measured into a 25 mL volumetric flask. To effectively remove high-molecular compounds, we added 1 mL each of Carrez I (15 g of potassium hexacyanoferrate (II) dissolved in 100 mL of distilled water) and Carrez II (30 g of zinc sulphate dissolved in 100 mL of distilled water) to the sample. The sample was stirred and gently slightly shaken and then allowed to stand for 10 min. After replenishing the flask with water, the sample was thoroughly stirred before being transferred in its entirety to a polypropylene centrifuge tube. The centrifuge operated at 10,000 rpm for 10 min at approximately 10 °C. The clear solution was swiftly and quantitatively transferred to an Erlenmeyer flask. Subsequently, 1 mL of the supernatant was filtered using a PVDF syringe filter (0.45 μm) into a chromatography vial.

Caffeine quantification in coffee was performed using an Alliance 2695 high-performance liquid chromatograph with a diode array detector (HPLC-DAD; Waters, Milford, MA, USA). This analysis was conducted on a Lichrospher RP-18 column (125 mm × 4 mm; 5 µm; Agilent Technologies, Santa Clara, CA, USA), including a pre-column (4 mm × 4 mm; 5 μm; Agilent Technologies), with the same packing material. Chromatographic analysis conditions: mobile phase—water and methanol HPLC (70:30; *v*/*v*), isocratic flow 0.9 mL/min, sample injection volume 20 μL, sample temperature 20 °C, column temperature 40 °C, UV detection at a wavelength of λ = 273 nm, total analysis duration 8 min. The identification of the test compound was carried out based on its retention time and a comparison between the acquired UV spectrum and the spectrum of the caffeine standard. The result was taken as an average of three parallel determinations, corrected for recovery for each analysis series.

The test method described above had undergone prior validation [48], and has received accreditation for compliance with requirements of EN ISO 17025 [49] by the Polish Centre for Accreditation. Our laboratory regularly participates in proficiency testing (PT), achieving satisfactory results.

### 4.5. Calculation of Polyphenol and Caffeine Content in a Coffee Serving

To calculate the polyphenol and caffeine content in a coffee serving, an individual serving size were employed for each type of coffee. The volume of purchased coffee servings was meticulously measured in the laboratory and, depending on the franchise coffee shops, was as follows range: 22–49 mL for espresso, 142–305 mL for caffè latte/latte macchiato, 140–320 mL for cappuccino, 155–360 mL for Americano and 245–420 mL for ice latte/latte frappe.

### 4.6. Statistical Analysis

The obtained analytical results underwent evaluation, including Dixon’s and Grubbs’s tests to eliminate outliers (with coarse errors). Subsequently, the data were subjected to comprehensive statistical analysis using the following software tools: Statistica version 6.0 (Statsoft, Inc., Tulsa, OK, USA) and Stata/SE 17.0. The results for polyphenol and caffeine content in coffee were expressed in two ways: as mg/100 mL of coffee brew and as mg per coffee serving. These values were presented as means, along with minimum and maximum values.

To evaluate the significance of differences between various coffee types, a one-way analysis of variance (ANOVA) was conducted. Subsequently, multiple comparisons were performed using Tukey’s test to determine significant differences between coffee types. Pearson’s linear correlation coefficient was use to evaluate the association between polyphenol and caffeine content. In all conducted statistical tests, a significance level of *p* ≤ 0.05 was applied to determine the presence of meaningful differences and relationships among the variables under examination.

## 5. Conclusions

This study showed a strong positive correlation between total polyphenol and caffeine content in all studied types of coffee from franchise coffee shops. It was concluded that the most important measure of the above-mentioned components supplied to the body with coffee is the serving size. Although espresso coffee had the highest total polyphenol and caffeine content per unit volume, given the serving size, up to ten times smaller than other coffees; the Americano serving provided the most polyphenols, while the ice latte/latte frappe serving had the highest caffeine content. In terms of the proportion of both bioactive compounds, it seems that the optimal coffee would be a serving of Americano, which contained the most polyphenols and the average caffeine content covers the minimum dose increasing alertness. While cups are a common measure of coffee intake in both scientific publications and everyday life, such a measurement method is not accurate for coffee bought in coffee shops, as most coffees are served in drinking vessels larger than a typical cup.

## Figures and Tables

**Table 1:** Content of polyphenols and caffeine in different types of coffee collected from franchise coffee shops (in mg/100 mL) *.

Components	Coffee Brew					*p*-Value
	Espresso	Caffè latte/Latte Macchiato	Cappuccino	Americano	Ice Latte/Latte Frappe	
total polyphenols	232.9 ± 63.9 ^c^ (114.3–343.1)	62.3 ± 10.8 ^ab^ (42.1–77.5)	66.9 ± 10.8 ^ab^ (51.9–85.8)	101.3 ± 45.2 ^b^ (46.0–194.1)	43.3 ± 16.2 ^a^ (21.3–72.9)	<0.005
gallic acid	56.3 ± 27.6 ^b^ (16.0–108.7)	33.8 ± 20.0 ^bc^ (2.3–74.9)	3.3 ± 1.4 ^a^ (1.8–5.2)	47.0 ± 47.6 ^b^ (1.8–128.6)	5.0 ± 4.3 ^ac^ (1.4–13.2)	<0.05
chlorogenic acid	45.6 ± 19.2 ^b^ (13.3–72.2)	4.9 ± 2.2 ^a^ (2.2–8.7)	14.4 ± 11.0 ^b^ (1.3–33.4)	43.8 ± 25.7 ^b^ (13.5–92.1)	5.6 ± 2.0 ^a^ (2.5–8.6)	<0.0001
caffeic acid	3.10 ± 1.79 ^b^ (0.34–5.46)	0.24 ± 0.17 ^a^ (0.08–0.65)	0.21 ± 0.10 ^a^ (0.10–0.40)	0.64 ± 0.54 ^a^ (0.09–1.60)	0.09 ± 0.09 ^a^ (0.02–0.26)	<0.0001
salicylic acid	1.23 ± 1.42 (0.08–4.99)	0.31 ± 0.32 (0.12–1.23)	0.68 ± 0.92 (0.08–2.80)	1.40 ± 1.50 (0.09–5.70)	0.24 ± 0.16 (0.08–0.60)	n.s.
epigallocatechin	0.67 ± 0.89 ^b^ (0.01–2.15)	0.07 ± 0.04 ^a^ (0.02–0.14)	0.09 ± 0.07 ^a^ (0.01–0.28)	0.75 ± 0.36 ^b^ (0.36–1.57)	0.06 ± 0.03 ^a^ (0.01–0.10)	<0.05
quercetin-3-*O*-rutinoside	0.15 ± 0.06 ^b^ (0.04–0.24)	0.05 ± 0.02 ^a^ (0.03–0.09)	0.08 ± 0.06 ^ab^ (0.02–0.23)	0.23 ± 0.11 ^c^ (0.12–0.48)	0.05 ± 0.02 ^a^ (0.02–0.09)	<0.05
kaempferol-3-*O*-glucoside	0.28 ± 0.17 (0.12–0.73)	0.33 ± 0.20 (0.09–0.58)	0.28 ± 0.77 (0.10–0.22)	0.27 ± 0.20 (0.12–0.82)	0.43 ± 0.21 (0.10–0.78)	n.s.
quercetin	0.05 ± 0.01 (0.03–0.06)	0.05 ± 0.01 (0.03–0.07)	0.05 ± 0.02 (0.03–0.10)	0.05 ± 0.01 (0.03–0.07)	0.05 ± 0.01 (0.03–0.07)	n.s.
quercetin-3-*O*-glucoside	5.95 ± 2.92 ^b^ (1.03–11.20)	4.69 ± 3.06 ^ab^ (1.39–9.06)	2.75 ± 0.88 ^a^ (1.52–4.01)	3.05 ± 1.85 ^ab^ (1.33–7.63)	4.46 ± 3.92 ^ab^ (0.85–11.03)	<0.05
kaempferol	1.64 ± 1.02 (0.63–3.76)	1.26 ± 1.03 (0.60–4.31)	1.38 ± 1.06 (0.53–4.31)	1.57 ± 0.86 (0.62–2.87)	1.14 ± 0.73 (0.53–2.62)	n.s.
caffeine	198.6 ± 68.3 ^b^ (109.9–370.4)	48.0 ± 16.7 ^a^ (20.5–88.9)	52.3 ± 17.5 ^a^ (27.1–96.7)	55.5 ± 26.9 ^a^ (33.2–133.6)	43.9 ± 14.2 ^a^ (21.8–70.6)	<0.0001

* Data are presented as the mean ± SD (min–max) with Tukey test *p*-value; means in rows followed by the same letter are not significantly different (*p* < 0.05); n.s. not significant statistically.

**Table 2:** Ratio of the caffeine content to selected polyphenols’ level *.

Components	Type of Coffee					*p*-Value
	Espresso	Caffè Latte/Latte Macchiato	Cappuccino	Americano	Ice Latte/Latte Frappe	
caffeine/total polyphenols	0.65 ± 0.19 ^a^ (0.32–0.96)	0.99 ± 0.48 ^ab^ (0.40–2.11)	0.78 ± 0.39 ^ab^ (0.32–1.81)	0.56 ± 0.34 ^a^ (0.21–1.37)	1.17 ± 0.42 ^b^ (0.49–1.85)	<0.05
caffeine/chlorogenic acid	4.3 ± 3.5 ^ab^ (1.8–13.4)	14.8 ± 10.1 ^c^ (3.6–39.5)	8.4 ± 9.4 ^ac^ (1.1–32.0)	1.5 ± 1.0 ^a^ (0.4–3.6)	9.2 ± 3.7 ^bc^ (3.7–14.6)	<0.05
caffeine/gallic acid	3.4 ± 2.1 ^a^ (1.5–6.9)	2.9 ± 3.4 ^a^ (1.0–13.6)	18.2 ± 11.3 ^b^ (7.5–46.0)	5.0 ± 9.5 ^a^ (0.3–34.3)	15.7 ± 10.4 ^b^ (3.2–29.9)	<0.05

* Data are presented as the mean ± SD (min–max) with Tukey test *p*-value; means in rows followed by the same letter are not significantly different (*p* < 0.05).

**Table 3:** Liquid chromatography gradient program.

Time (min)	Flow (mg/mL)	% A	% B
Initial	1.00	95	5
23	1.00	50	50
28	1.00	80	20
29	1.00	95	5
Total runtime: 38 min			

## Data Availability

Data are contained within the article.
